# SmvA is an important efflux pump for cationic biocides in *Klebsiella pneumoniae* and other Enterobacteriaceae

**DOI:** 10.1038/s41598-018-37730-0

**Published:** 2019-02-04

**Authors:** Matthew E. Wand, Shirin Jamshidi, Lucy J. Bock, Khondaker Miraz Rahman, J. Mark Sutton

**Affiliations:** 1 Public Health England, National Infection Service, Porton Down, Salisbury, Wiltshire SP4 0JG UK; 2 School of Cancer and Pharmaceutical Science, King’s College London, London, SE1 9NH UK

## Abstract

The multidrug resistant (MDR) opportunistic pathogen *Klebsiella pneumoniae* has previously been shown to adapt to chlorhexidine by increasing expression of the MFS efflux pump *smvA*. Here we show that loss of the regulator SmvR, through adaptation to chlorhexidine, results in increased resistance to a number of cationic biocides in *K. pneumoniae* and other members of the Enterobacteriaceae. Clinical Enterobacteriaceae isolates which lack *smvA* and *smvR* also have an increased susceptibility to chlorhexidine. When *smvA* from *Salmonella* and *K. pneumoniae* are expressed in *Escherichia coli*, which lacks a homologue to SmvAR, resistance to chlorhexidine increased (4-fold) but plasmid carriage of *smvA* alone was detrimental to the cell. Challenge of *K. pneumoniae* with chlorhexidine and another cationic biocide, octenidine, resulted in increased expression of *smvA* (approx. 70 fold). Adaptation to octenidine was achieved through mutating key residues in SmvA (A363V; Y391N) rather than abolishing the function of SmvR, as with chlorhexidine adaptation. Molecular modelling was able to predict that octenidine interacted more strongly with these mutated SmvA forms. These results show that SmvA is a major efflux pump for cationic biocides in several bacterial species and that increased efflux through SmvA can lead to increased chlorhexidine and octenidine tolerance.

## Introduction

With the rise in the number of infections caused by multi-drug resistant (MDR) pathogens, the pressure on antibiotic usage and infection prevention by other means has increased. This includes the use of biocides for disinfection of surfaces, medical devices and skin prior to operations. There is concern that the indiscriminate use of biocides is able to select for increased tolerance to particular biocides and also cross-resistance to antibiotics^1–4^. Indeed, overexpression of MDR efflux pumps such as *acrAB-tolC* in *Escherichia coli*^5^, *mexCD-oprJ* in *Pseudomonas aeruginosa*^6^ and *smeDEF* in *Stenotrophomonas maltophilia*^7,8^ has been linked to increased biocide tolerance.

The bisbiguanide chlorhexidine is widely used in clinical environments for many applications such as in mouth washes and wound dressings. Increased chlorhexidine susceptibility has been linked to deletion of a number of efflux pumps including *aceI* in *Acinetobacter baumannii*^9^ and *cepA* in *Klebsiella pneumoniae*^10^. Previously, we have shown that increased tolerance of chlorhexidine in *K. pneumoniae* is associated with increased expression of the efflux pump *smvA* through deletions in the adjacent divergently-transcribed gene termed *smvR*^11^.

SmvA was originally described as a chromosomally encoded major facilitator superfamily (MFS) efflux pump implicated in methyl-viologen efflux in *Salmonella*^12,13^. SmvR, like other TetR-like repressors is characterised by a HTH DNA binding domain^14^. The TetR family of regulators include AcrR, BetI and EnvR^15,16^, as well as those which have been implicated in biocide resistance, such as QacR from *Staphylococcus aureus* and AdeN from *A. baumannii,* which regulate the efflux pumps QacA and AdeIJK, respectively^17,18^. QacR has been shown to interact with cationic compounds such as dequalinium, chlorhexidine and ethidium^19,20^ by utilising four glutamate residues lining the drug-binding interface to bind to the cationic compounds^21^. QacR regulates the expression of *qacA* and also its own expression by binding to specific palindromic repeats in the promoter region^15,16^.

The aim of this study was to investigate the importance of SmvAR in tolerance to cationic biocides including chlorhexidine. To show this, we have examined whether *K. pneumoniae* strains, which have mutations in *smvR,* have decreased susceptibility to a range of cationic biocides, not just chlorhexidine. We also explored the importance of SmvAR in other Enterobacteriaceae (namely *Citrobacter sp., Enterobacter sp*. and *Salmonella enterica* serovar Typhimurium) in providing increased tolerance to chlorhexidine. We show that expression of *smvAR* in *E. coli* (an organism which appears to lack these genes) leads to decreased chlorhexidine susceptibility and that expression of *smvA* in *K. pneumoniae* is upregulated following chlorhexidine challenge. Computer modelling was employed to predict that chlorhexidine is able to bind to SmvA and identified specific residues that are theorised to be involved in the interaction of SmvA with chlorhexidine. Overall this demonstrated a clear role of SmvAR in mediating increased resistance to a range of cationic antiseptics.

## Results

### SmvA acts as a more general cationic biocide efflux pump in *K. pneumoniae*

We utilised transposon mutants in *smvA* and *smvR* derived from the *K. pneumoniae* strain MKP103^22^ to understand more about the function of SmvAR and its relationship with biocide resistance. These transposon mutants had a T30 transposon (a derivative of Tn5 carrying a chloramphenicol resistance gene) inserted into either *smvR* or *smvA*. There were three transposon mutants in *smvR* (clones KP05926 (∆*smvR1*), KP05927 (∆*smvR2*), KP05928 (∆*smvR3*)) and a single transposon mutant in *smvA* (KP05925 (∆*smvA*)). All transposon mutants were whole genome sequenced and details of the position of transposon insertion are provided (Supplementary Table 1). MIC/MBCs were determined against a range of biocides (Table 1). For several cationic biocides, including chlorhexidine, CPC, CTAB, and cetrimide, the absence of SmvR led to a decrease in susceptibility (≥2-fold) relative to MKP103. For ∆*smvA* there was a 2-fold increase in susceptibility for chlorhexidine, cetrimide, CTAB and HDPCM. Transposon disruption of either *smvR* or *smvA* appeared to have no impact on antibiotic resistance for all antibiotics tested (Supplementary Table 2). However, MKP103 contains several antibiotic resistance markers including aminoglycoside resistance genes (*aadA2*, *aph(3*′*)-Ia, aac(6*′*)-Ib*), fluoroquinolone resistance caused by mutations in GyrA (S83I) and ParC (S80I) as well as colistin resistance, which we hypothesise is caused by a previously undescribed mutation in PhoQ (Y265C). Therefore, it is plausible that any effects caused by loss of SmvA or SmvR function are masked by high level resistance mediated by pre-existing antibiotic resistance mechanisms.

**Table 1:** MIC (A) and MBC (B) values for *smvA* and *smvR* mutants compared to the wildtype MKP103.

	CHD	MV	BAC	BEC	CPC	CTAB	DDAB	ALX	DQC	TRC	ETH	OCT	CET	HDPCM	NaDCC	VRK	PAD	H_2_O_2_	GLT
(A)																			
MKP103	64	>1024	32	64	8–16	64	16	2–4	256	2–4	6.25	4–8	0.004–0.007	16	125–250	0.5	0.04	0.005	0.39
∆*smvA*	16–32	>1024	32	64	8	64	16	4	256	2	6.25	4–8	0.002–0.004	8	250	0.5	0.04	0.01	0.39
∆*smvR1*	128–256	>1024	32	64	32	256	16	4	256	2	6.25	4	0.03	64	250	0.5	0.04	0.01	0.39
∆*smvR2*	128–256	>1024	32	32–64	64	256	16	4	256	2	6.25	4–8	0.03	64	250	0.5	0.04	0.005	0.39
∆*smvR3*	128–256	>1024	32	64	32	256	16	4	256	2	6.25	4–8	0.03	64	250	0.5	0.04	0.005	0.39
(B)																			
MKP103	64	>1024	32	64	8–16	64–128	16	4	>512	4	6.25	4–8	0.007–0.015	16	125–250	0.5	0.04	0.005	0.39
∆*smvA*	32	>1024	32	64	8	64	16	4	>512	2	6.25	8	0.002–0.004	8	250	0.5	0.04	0.01	0.39
∆*smvR1*	512	>1024	32	64	32–64	256	16	4	>512	4	6.25	8	0.03	64	250	0.5	0.04	0.01	0.39
∆*smvR2*	512	>1024	32	64	64–128	256	16	4	>512	2	6.25	8	0.03	64	250	0.5	0.04	0.01	0.39
∆*smvR3*	512	>1024	32	64	32–64	256	16	4	>512	2	6.25	8	0.03	64	250	0.5	0.04	0.005	0.39

All values are in mg/L except for VRK (% Working concentration), CET, ETH, PAD, H_2_O_2_, GLT (all %) and NaDCC (ppm). Abbreviations used: chlorhexidine digluconate (CHD), methyl-viologen (MV), benzalkonium chloride (BAC), benzethonium chloride (BEC), cetylpyridinium chloride (CPC), cetyltrimethylammonium bromide (CTAB), didecyldimethylammonium bromide (DDAB), alexidine dihydrochloride (Alex), dequalinium chloride hydrate (DQC), Triclosan (TRC), ethanol (ETH), octenidine hydrochloride (OCT), cetrimide (CET), hexadecylpyridinium chloride monohydrate (HDPCM), sodium dichloroisocyanurate (NaDCC), virkon (VRK), peracetic acid (PAD), hydrogen peroxide (H_2_O_2_) and glutaraldehyde (GLT). All MIC/MBCs were performed in triplicate.

**Figure 1:**
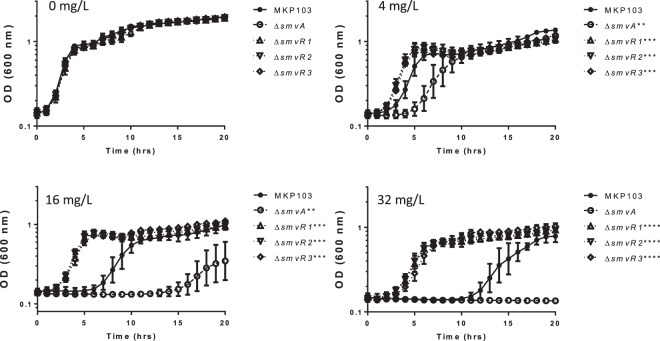
Growth analysis of MKP103 ∆*smvA* and ∆*smvR* clones in the presence of chlorhexidine (CHD). Strains of *K. pneumoniae* were grown for 20 hrs (up to MIC measurement time) in different concentrations of chlorhexidine. The data presented are the means of four biological independent replicates. Significant differences in growth of MKP103 ∆*smvA* and MKP103 ∆*smvR*(1–3) relative to MKP103 are shown in the individual graph legends.

Due to there being a modest difference (2–4 fold) in the MIC values for chlorhexidine of the transposon mutants relative to MKP103, MIC experiments were repeated but the OD_600_ was recorded every hour to generate a growth profile. For chlorhexidine, as the concentration of biocide increased the differences in growth rate between the mutants and the wild-type (MKP103) became more significant. The *smvR* transposon mutants reached maximal OD_600_ values more rapidly in the presence of increased concentrations of chlorhexidine, whilst ∆*smvA* growth rate was retarded compared to MKP103 at the concentrations shown (Fig. 1).

### SmvA is upregulated in response to chlorhexidine

Although we have recently shown that *smvA* is upregulated in mutants which have a non-functional SmvR^11^, we wanted to ascertain whether *smvA* transcript levels are altered in direct response to chlorhexidine. Therefore, *K. pneumoniae* strain MGH 78578, which contains full length *smvA* and *smvR* genes, was challenged with sub-MIC (8 mg/L) and lethal (128 mg/L) concentrations of chlorhexidine. The same strain was also challenged with sub-MIC (1 mg/L) and lethal (16 mg/L) concentrations of another cationic biocide, octenidine. This was because octenidine is increasingly used as a substitute for chlorhexidine and, unlike chlorhexidine, MIC/MBC values for octenidine for the *smvA*/*smvR* transposon mutants showed no change relative to MKP103. At the sub-MIC level for chlorhexidine, there was a significant upregulation of *smvA* (70-fold) and *smvR* (14-fold). At the lethal level there was upregulation of *smvA* (15-fold) and *smvR* (3.5 fold). Perhaps surprisingly, given the lack of change in MICs following disruption to either *smvR* or *smvA*, after exposure to octenidine, there were similar levels of upregulation for both *smvA* and *smvR* as seen after challenge with chlorhexidine at both the sub-MIC and lethal concentrations (Supplementary Fig. S1).

### Expression of *smvA* in *E. coli* increases tolerance to chlorhexidine

**Figure 2:**
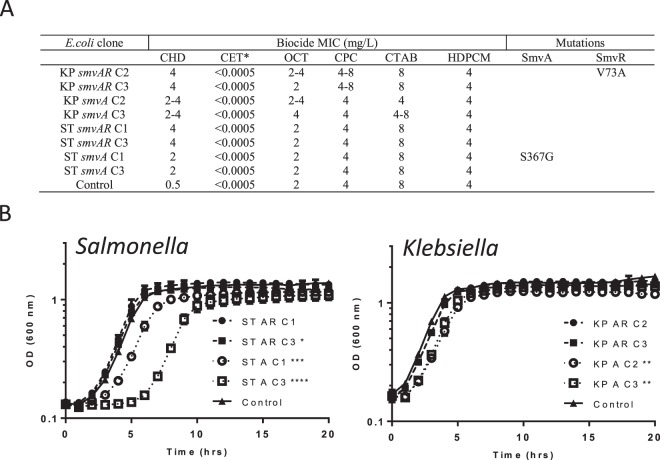
Introduction of *smvA* into *E. coli* leads to increased resistance to chlorhexidine. MIC values to cationic biocides (**A**) and growth profiles (**B**) for *E. coli* expressing plasmids containing *smvA* or *smvAR* from *S. enterica* or *K. pneumoniae*. For *E. coli* clones KP indicates the fragment was cloned from *K. pneumoniae* and ST from *S. enterica* serovar Typhimurium. *smvAR* indicates that the fragment contains full length *smvA* and *smvR* including their promoters and *smvA* indicates that the fragment contains a full length *smvA* including its promoter. C2 indicates clone number 2 etc. Coding mutations found in various fragments are indicated. Control indicates plasmid only. Growth curves are the means of three independent repeats and statistical significance between clones and the control is shown where appropriate. All MIC values are given as mg/L except CET (%). For abbreviations see Table 1.

Blast searches revealed that *E. coli* contained no homologue for SmvAR and the majority of *E. coli* isolates tested had an MIC value of 0.5–1 mg/L to chlorhexidine (Supplementary Table 3), which is lower than *Klebsiella* (typically 16–32 mg/L) or other SmvAR containing members of the Enterobacteriaceae family tested. To test whether expression of *smvAR* from other Enterobacteriaceae would increase the resistance to chlorhexidine in *E. coli*, *smvA* and the *smvAR* region from *K. pneumoniae* MGH 78578 and *Salmonella enterica* serovar Typhimurium SL1344 were cloned into TOPO Vector pCR 2.1 and transformed into *E. coli* TOP10 (K-12) cells. Sequence analysis of several clones indicated that there were often mutations in the *smvA* sequence despite the original PCR product not showing any such mutations. Two clones, each containing either *Salmonella smvA* or *smvAR* or *Klebsiella smvA* or *smvAR,* were selected for further MIC testing, including, where possible, clones with no SNPs in *smvA* or *smvR*. All clones tested showed increased (4–8 fold) MIC values (2–4 mg/L) for chlorhexidine relative to plasmid only control (0.5 mg/L), but not for other cationic disinfectants tested (Fig. 2A). *E. coli* expressing *Salmonella smvAR* consistently gave 2-fold higher MIC values for chlorhexidine relative to clones expressing *smvA* alone. The growth of strains expressing *K. pneumoniae*, but especially for *S. enterica smvA* only, was also significantly reduced relative to SmvAR containing strains (Fig. 2B), potentially indicating that the expression of *smvA* without its regulator was more toxic to *E. coli*.

### SmvR mutations are observed in other members of the Enterobacteriaceae upon chlorhexidine exposure and these also impact on cationic biocide resistance

**Figure 3:**
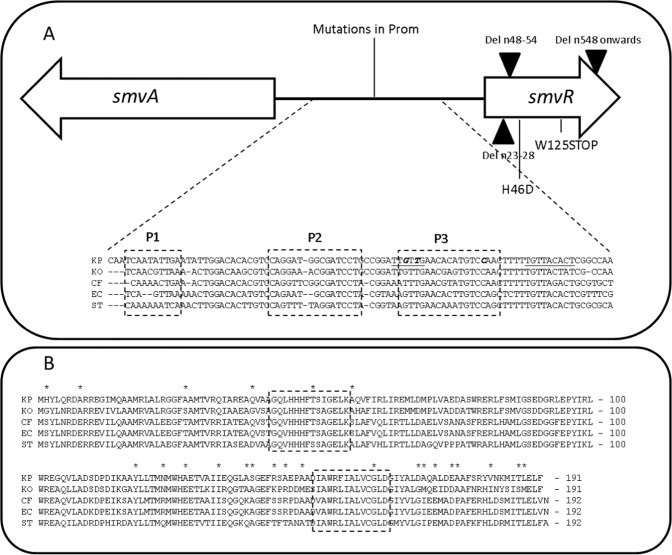
Sequence analysis for the *smvA* and *smvR* promoter region (**A**) and protein sequence for SmvR (**B**) between select members of the Enterobacteriaceae. Consensus sequences from *K. pneumoniae* (KP), *K. oxytoca* (KO), *C. freundii* (CF), *E. cloacae* complex (EC) and *S. Typhimurium* (ST) were aligned. Potential DYAD repeats in *K. pneumoniae* (P1 to P3) are indicated and mutations after CHD adaptation in *K. pneumoniae* are indicated in the gene and the promoter (bold italic). The −10 and −35 promoter regions are also indicated (underlined). (**B**) Highly conserved regions in the Enterobacteriaceae in the SmvR protein are indicated in boxes. One of the mutations (H46D) occurs in the N-terminal conserved region within the predicted DNA binding region. *indicates natural sequence variation in *K. pneumoniae*.

Whilst mutations were observed in *K. pneumoniae smvR* following adaptation to chlorhexidine, we wanted to understand whether similar mutations were found in other members of the Enterobacteriaceae upon exposure to chlorhexidine. Therefore, strains of *Klebsiella oxytoca*, *Citrobacter freundii*, *Enterobacter* sp. and *S. enterica*, were exposed to increasing concentrations of chlorhexidine. Several *K. pneumoniae* strains were also adapted alongside as a control. Results showed that the *Klebsiella* species were most able to adapt, with 5/7 and 4/7 strains surviving in concentrations of 128 mg/L CHD for *K. pneumoniae* and *K. oxytoca* respectively. Mutations observed following chlorhexidine adaptation are described in Table 2. This shows the presence of additional *K. pneumoniae* mutations in *smvR*, along with several mutations in the *smvAR* promoter region, which had not been described in our previous study^11^. There were no mutations in PhoPQ identified as found previously^11^ but several strains had deletions in *mgrB*, the *phoP* regulator gene. Mutations in *mgrB* were also found in *K. oxytoca* but no other strains showed genetic changes in the *phoPQ* controlled system. *SmvR* mutations were also found in *Salmonella*, *C. freundii* and *K. oxytoca* strains but not *Enterobacter* spp. Other mutations of interest include genetic changes in another TetR-family transcriptional regulator *ramR*, members from the BamABCDE complex involved in OM biogenesis e.g. *bamE* from *Salmonella*, and the MltA-interacting protein *mipA*. Mutations in *mipA* and *bamD* have been previously observed in *K. pneumoniae*^11^. For mutations in *smvR*, all mutations in non-*Klebsiella* species were found in the C-terminal ligand binding region; in *Klebsiella* mutations were either in the promoter or in the N-terminal DNA binding region. However, we have previously observed mutations throughout the gene (Fig. 3A)^11^. Therefore, adaptation to chlorhexidine thorough mutations in *smvR* is not exclusive to *K. pneumoniae*, but is observed in diverse members of the Enterobacteriaceae. For those adapted strains from the other members of the Enterobacteriaceae with mutations in SmvR, there was an increase in tolerance to chlorhexidine and several other cationic biocides. This suggests that SmvA has a similar role in mediating tolerance to cationic biocides in several members of the Enterobacteriaceae.

**Table 2:** Mutations observed after chlorhexidine adaptation in different members of the Enterobacteriaceae.

Organism	Strain	SmvR mutation	PhoPQ pathway mutation	Other mutation
*K. pneumoniae*	20	SNP in promoter	∆*mgrB*	
		SNP in promoter		
	6	Del nucleotides 48–54		RamR E7STOP WbaP Q386K
	16		MgrB W20STOP	SurA V32L
	19	SNP H46D	∆*mgrB*	
	CFI_141-KPC			WbaP D445E
*K. oxytoca*	7227	SNP in promoter		CcmF F413V
	7555			LpxM Del nucleotide 105 (truncated 37 aa protein)
	5490		∆*mgrB*	Several genes around *mgrB* were also deleted.
	CFI_80_KPC			RamR N56I
				RamR 1 bp del (truncated 123 aa protein)
*C. freundii*	7556			OmpH Del nucleotide 270 (truncated 123 aa protein)
	NCTC 9750	SNP L182Q		Late control protein D (SNP in promoter)
	8	Transposon insertion		YbaL T373A
				BamA D512Y
*E. cloacae*	7558a			AmpG L309Q
	7558b			YebN insertion GGCTTCT after nucleotide 309
				MipA insertion G after nucleotide 381
*E. aerogenes*	CFI_16_OXA48			LptC G153R
*S. enteritidis*	FW1401	Duplication aa 153–157		BamE W73STOP
*S. arizonae*	FWE06			BamE Del nucleotide 47 (truncated 15 aa protein)

Investigation of *Klebsiella*, *Salmonella*, *Citrobacter* and *Enterobacter* clinical strains from our collection, which had been whole genome sequenced, showed that a few strains lacked *smvAR* or had disrupted *smvA* genes. These strains showed reduced susceptibility to chlorhexidine and other cationic biocides (cetrimide, CPC, CTAB and HDPCM) (Table 3).

**Table 3:** Variants in SmvAR and their effect on biocide tolerance.

Strain	Biocide MIC (mg/L)						SmvAR status	
	CHD	CET*	OCT	CPC	CTAB	HDPCM	SmvA	SmvR
***Enterobacter***								
BS35	16	0.0039	4	32	32	16	WT	C-terminal Extension
BS92	8	0.0009	2	8	16	16	WT	WT
CFI_002_CRCN	2	≤0.0005	2	8	8	8	Absent	Absent
CFI_004_KPC	1	≤0.0005	1	4	4	2	Disrupted	WT
***Salmonella***								
FWE 1401	8	0.0039	2	16	32	16	WT	WT
FWE 1401 CHD	512	0.07	2	32	64	16	WT	Insertion
***Citrobacter***								
CK 51	2	0.0009	1	1	8	4	Disrupted	WT
CK 54	2	0.0009	1	1	8	4	Disrupted	WT
NCTC 9750	8	0.0009	2	2	8	8	WT	WT
NCTC 9750 CHD	32	0.0019	2	2	16	8	WT	Mutation
8	32	0.0039	2	2	32	16	WT	WT
8 CHD	256	0.015	4	4	64	32	WT	Transposon insertion
***K. pneumoniae***								
BS26	8–16	≤0.0005	4	4	8	4	Disrupted	WT
M403	≤0.5	≤0.0005	2	4	8	1	Absent	Absent
M635	≤0.5	≤0.0005	2	4	8	1	Absent	Absent
M665	≤0.5	≤0.0005	2	2	4	2	Absent	Absent
KP 115	1	0.0019	2	4	16	4	Absent	Absent
6	32	0.0009	4	4	16	4	WT	WT
6 CHD	128	0.007	4	64	128	64	WT	Deletion
19	32	0.0018	4–8	16	32	16	WT	WT
19 CHD	64	0.0018	8	16	32	16	WT	Mutation
20	32	0.0009	8	4	8	4	WT	WT
20 CHD	64	0.003	4–8	8	16	32	Mut in prom	Mut in prom
***K. oxytoca***								
0552	4	0.007	4	8	32	16	Absent	Absent
7227	16	0.015	4	8	128	16	WT	WT
7227 CHD	64	0.015	8	32	128	64	Mut in prom	Mut in prom

WT indicates that the sequence is full length. For list of abbreviations see Table 1. CHD indicates strains which have been adapted to chlorhexidine. *Value is given as %.

Further sequence analysis of the shared promoter region for *smvAR* revealed the presence of three palindromic DYAD motifs which are typical for Tet-repressors and have been described for QacR^15^. In particular one palindromic motif (P3) was highly conserved between all species analysed (Fig. 3A). Indeed, SNPs in the *smvAR* promoter region, of *Klebsiella* strains after adaptation to chlorhexidine, affected the symmetry of palindromic sequence P3, which indicated that this is a likely SmvR binding site. Alignment of the 138 bp intergenic region for *smvA* and *smvR* for all *K. pneumoniae* strains in our database (approx. 90) showed that the sequence is highly conserved with most changes being sequence-type associated, although several strains have an affected palindromic motif (P1) (Supplementary Fig. S2). These strains do not appear to have a noticeable change in chlorhexidine MIC, possibly indicating that P1 is not important for SmvR binding. There are sigma^70^ −35 and −10 regions predicted for *smvR* but not for *smvA* which may indicate that under normal conditions *smvA* expression may be downregulated. Analysis of the SmvR sequence from several members of the Enterobacteriaceae showed areas of conserved homology, in particular two areas highlighted in Fig. 3B and Supplementary Fig. S3, one in the predicted DNA binding N-terminal domain and one in the C-terminal substrate recognition domain which may indicate that similar substrates are recognised by the SmvR proteins from various different species.

### Chlorhexidine is able to bind to SmvA through specific residues

**Figure 4:**
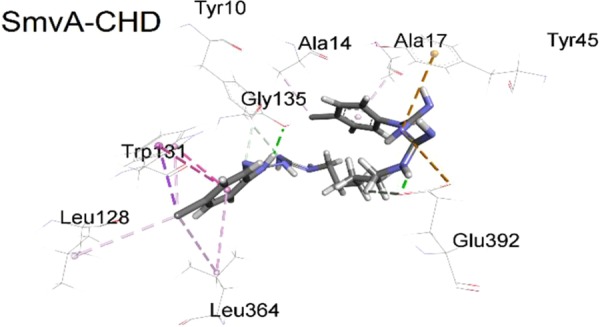
Molecular level interactions of chlorhexidine (CHD) within the binding pocket of *K. pneumoniae* SmvA. CHD showed hydrogen bond interactions with GLU392 and TYR10, and a range of hydrophobic interactions with LEU128, LEU364, ALA17 and TRP131.

To understand potential interactions between SmvA and chlorhexidine, homology modelling and molecular docking studies were employed. These were able to show that theoretically chlorhexidine is able to interact with the central channel of SmvA (Fig. 4). A 100 ns molecular dynamics (MD) simulation provided information about the interaction of chlorhexidine with the binding pocket in SmvA. The complex remained stable during the course of the simulation largely due to the formation of hydrogen bonds and hydrophobic interactions with different residues. Hydrogen bond analysis showed that there are two permanent hydrogen bonds for SmvA-Chlorhexidine centred at residue Q392.

### SmvA mutations occur in response to octenidine adaptation

**Figure 5:**
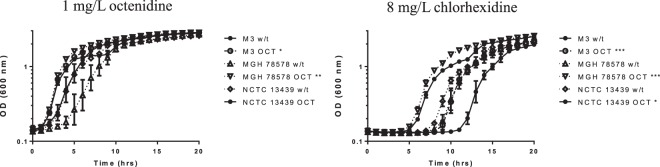
Growth of octenidine adapted *K. pneumoniae* strains in sub-lethal levels of octenidine and chlorhexidine. Growth curves are the means of three independent repeats and the statistical significance in growth rate between octenidine adapted (OCT) and respective parental (w/t) strains is shown.

Given the result that *smvA* was upregulated following exposure to octenidine, we sought to understand whether SmvA is also involved in the adaptive mechanism(s) associated with increased octenidine resistance. Strains of *K. pneumoniae* were adapted to octenidine in a similar manner to chlorhexidine. Several strains were able to survive in concentrations of 32–64 mg/L octenidine. When these were tested for increased MIC/MBC to octenidine there was only a two-fold increase for adapted strains compared to the parental strains (Table 4). There was also no noticeable cross-resistance to chlorhexidine or other cationic biocides. However, in general, these mutants again showed a significantly reduced doubling time, compared to their parental strains, in the presence of sub MIC-levels of either octenidine or chlorhexidine (Fig. 5), indicative of greater fitness under these conditions.

**Table 4:** Mutations observed and MIC values for cationic biocides following octenidine adaptation in *K. pneumoniae* strains. w/t implies parental non-adapted strain and OCT means strain has been adapted to octenidine.

Strain	Mutations after exposure		MIC Level (mg/L)					
	SmvA	Other	CHD	CET*	OCT	CPC	CTAB	HDPCM
NCTC 13443 w/t	—	—	16–32	0.0015	2	8	16–32	8
NCTC 13443 OCT SCV	A363V	ArnA A504V 50S ribosomal protein L1 Q80STOP	16	0.0015	4	8–16	32	8
NCTC 13443 OCT LCV	L364Q		32	0.0015	4	8–16	16	8
NCTC 13438 w/t	—	—	16–32	0.0015	2	8	16–32	8
NCTC 13438 OCT	A363V		16	0.0015	4	8	16	8
MGH 78578 w/t	—	—	16	0.0007–0.0015	2	8	8–16	8
MGH 78578 OCT	Y391N		16–32	0.0015	4	8	8–16	8
M3 w/t	—	—	8	0.0003–0.0007	2	4	8	4–8
M3 OCT	A363V	WcaJ L33STOP NlpD L37Q	16	0.0007	4	4–8	8–16	8
NCTC 13439	—	—	16–32	0.0015–0.003	2	16	16–32	16
NCTC 13439 OCT	A363T	SNP in promoter for *sulA*	16–32	0.003–0.007	4	16	16–32	16–32

*CET values are given as %. For abbreviations see Table 1.

**Figure 6:**
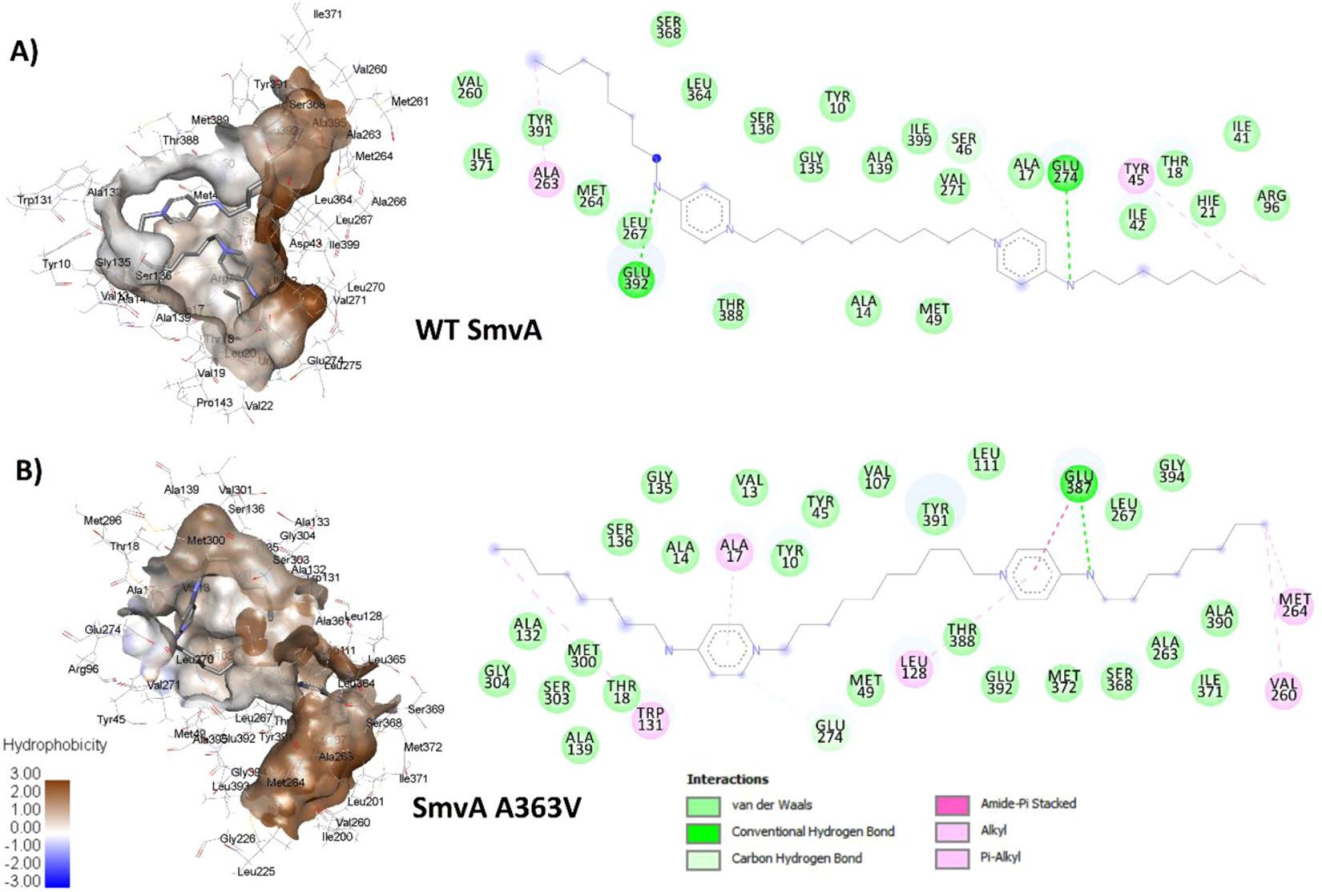
Post-MD simulation 3D and 2D interaction analysis of wild type SmvA and mutant A363V SmvA complexes with octenidine.

Analysis of the whole genome sequences for these adapted strains showed that they all contained a SNP in *smvA* with the majority (4/6 strains) containing a mutation which changed amino acid 363. The other mutations which were found were L364Q and Y391N in one strain each. All these mutations likely affect the binding and positioning of the L364 and E392 residues, which were flagged as important for hydrogen bond formation in chlorhexidine binding (Fig. 4). Molecular docking of octenidine with the wild type SmvA and the A363V and Y319N mutants showed good binding energy −50.62, −49.43 and −47.77 kcal/mol respectively. However, the mutant A363V showed slightly better energy of binding (−49.43 kcal/mol) compared to Y319N mutant (−47.77 kcal/mol), and the A363V mutant complex also displayed better ChemScore value (48) compared to both wild type (45) and Y391N mutant (43). A 100 ns MD simulation was carried out to investigate the molecular level interactions within the dynamic system, and the post-MD simulation analysis showed superior relative binding free energy values for both mutant A363V (−58.65 kcal/mol) and Y319N (−57.84 kcal/mol) complexes compared to the wild type (−45.31 kcal/mol) complex (Table 5). The 2D interaction analysis of the complexes after the MD simulation highlighted notably stronger interactions, both electrostatic and hydrophobic, with the mutant A363V structure compared to the wild type structure (Fig. 6) and Y319N mutant (Supplementary Fig. S4), which supports the energy values obtained for the complexes. The energy values and interaction analysis suggest both wild type and mutant SmvA have a sufficient specificity and selectivity to octenidine that can form stable complexes with good energy of binding, which consequently could lead to transport of octenidine across the channel.

**Table 5:** Post-MD simulation average energy contributions (kcal/mol) observed for different OCT-SmvA complexes.

Complex	MGH 78578 SmvA-OCT	A363V SmvA-OCT	Y391N SmvA-OCT
ΔE_ele_	12.07 (9.32)	41.69 (12.25)	−49.14 (7.77)
ΔE_vdw_	−59.36 (2.62)	−67.93 (2.12)	−57.49 (2.40)
ΔE_sur_	−9.72 (0.31)	−10.18 (0.24)	−8.29 (0.15)
ΔE_sol_	1.98 (10.46)	−32.42 (10.42)	48.79 (7.98)
ΔG_PB_	**−45.31** (**4.59)**	**−58.65** (**5.68)**	**−57.84** (**3.35)**
ΔG_GB_	**−53.54** (**2.78)**	**−66.92** (**3.06)**	**−57.14** (**2.48)**

## Discussion

This study has shown that SmvA is an important efflux pump for chlorhexidine and other cationic biocides in *K. pneumoniae* and other members of the Enterobacteriaceae tested. This is also the first study to describe a potential resistance mechanism for *K. pneumoniae* to octenidine and suggests that adaptation to chlorhexidine and octenidine occurs through a common mechanism (SmvAR). With increasing use of antiseptics, particularly to help combat the spread of MDR infections, there has been a lack of surveillance to look for the prevalence of potential antiseptic resistance mechanisms^23^. We and others have shown that bacterial strains of *K. pneumoniae* and *S. aureus* isolated in the last few years have higher MIC values to chlorhexidine and octenidine than strains isolated before the widespread use of these antiseptics^24,25^. Therefore, it is possible that bacteria are becoming more tolerant to these antiseptics by mechanisms highlighted in this study.

The addition of *smvAR* from *Salmonella* and *Klebsiella* into *E. coli* increased tolerance to chlorhexidine; but when the regulator was not present, *smvA* was likely to be highly expressed and caused a growth defect in the cell. Mutations were often observed in *smvA* or its promoter, which are likely minimising the damaging effect of overexpressed *smvA* on cell growth. Overexpression of another native MFS pump in *E. coli* (*mdtM*) has also been shown to be energetically costly^26^. *Klebsiella* strains were able to tolerate high levels of *smvA* expression (above 50-fold) in sub-MIC levels of chlorhexidine and those strains which had mutations in SmvR following chlorhexidine adaptation, generally had a constitutive 10–25 fold increase in *smvA* expression^11^. However, qPCR analysis in this study was performed after exposure to chlorhexidine for 20 mins and therefore the long-term effect of highly increased expression of *smvA* is unknown. Several adapted strains from a previous study were found to have additional mutations, such as those observed in PhoPQ, which may help to stabilise the membrane and compensate for the increased levels of SmvA^11^. PhoPQ mutations were found to contribute towards colistin resistance, but on their own did not increase resistance to chlorhexidine^11,27^.

Mutations in palindromic repeats present in the promoter of *smvA* identified after chlorhexidine adaptation presumably affect the binding of SmvR to the *smvA* promoter, leading to overexpression of *smvA*. The *E. coli* efflux pump, MdfA, which is thought to efflux specific divalent cationic compounds like chlorhexidine, is naturally present in a closed state and undergoes a conformation change upon binding of specific compounds^28,29^. Therefore, SmvA could also exist in two states, which depend on the presence of cationic compounds. Efflux systems may be recruited in response to environmental and external stress^30^ and the inactivation of the “stress response” in *P. aeruginosa* led to increased susceptibility to several antimicrobials^31^, highlighting its importance in antimicrobial resistance. We have yet to understand the main function of SmvA in *K. pneumoniae* and whether it is specific for cationic compounds or part of a less-specific stress response. We also do not understand whether SmvR is able to respond to a variety of external stimuli and regulate multiple genes rather than just *smvA*. Several *K. pneumoniae* strains from the Murray collection, which were isolated before the widespread use of many cationic biocides, contain *smvA* genes with no sequence deviation from modern day clinical isolates^24^, suggesting that this MFS pump has other naturally-occurring substrates. In *Klebsiella*, it is possible that multiple pumps are involved in the efflux of chlorhexidine. The finding of mutations in *ramR*, the indirect regulator of *acrAB*, in multiple strains after adaptation to chlorhexidine suggests a role for *acrAB* in chlorhexidine efflux. Other studies have indicated that specific pumps *cepA* and *kpnGH* are important in chlorhexidine efflux^10,32^. However, we have never observed mutations in either of these pumps following chlorhexidine adaptation and all strains of Enterobacteriaceae which lacked *smvAR* and were highly susceptible to chlorhexidine appeared to contain full length *cepA* and *kpnGH* equivalent genes.

Although we have yet to find clinical isolates of *Klebsiella* sp. with mutations in SmvR, we have recently identified a naturally occurring *smvR* deletion in *Proteus mirabilis* with elevated MICs for chlorhexidine (Pelling *et al*., manuscript in preparation). It is, however, clear that clinical isolates lacking *smvAR* are more susceptible to the action of cationic biocides. A homologue of *smvA* and its regulator has been found on a genomic island *SmarGI1-1*, containing the carbapenemase *sme-2* in *Serratia marcescens*^33^, which can be transferred between *Serratia* strains^34^. This raises the possibility of transfer of SmvAR to other species, which may contribute to more widespread and increased chlorhexidine tolerance in clinical pathogens. Transfer of *smvAR* to different species and/or *smvR* deletion in clinical strains is therefore, likely to have a significant impact on bacterial resistance to multiple cationic biocides.

This study has highlighted the importance of the efflux pump SmvA in generating increased resistance to chlorhexidine, octenidine and other cationic biocides in *Klebsiella* and other Enterobacteriaceae. Increased *smvA* expression, whether as a response to chlorhexidine or octenidine exposure, or due to possible plasmid transfer, may cause a concern for the effectiveness of infection prevention procedures in the clinic. Monitoring *smvAR* mutations in clinical strains would provide an early warning of potential reductions in susceptibility to critical antiseptics, either directly by SmvA overproduction or in association with other resistance mechanisms.

## Materials and Methods

### Growth of bacterial strains and adaptation to biocides

All strains were grown in tryptic soy broth (TSB) with aeration or on tryptic soy agar at 37 °C unless otherwise stated. Transposon mutants from *K. pneumoniae* MKP103 have been described previously^22^. All transposon mutants were whole genome sequenced prior to use. Adaptation to biocides was carried out as described previously^35^ except that the starting concentrations were 4 mg/L chlorhexidine up to a final concentration of 128 mg/L. For octenidine the starting concentration was 1 mg/L up to 64 mg/L.

### Determination of MIC/MBC

Broth-Microdilution MIC analysis was performed as described previously^36^. All MIC’s were performed in TSB media using polystyrene 96 well plates (Corning, Flintshire, UK) except for the polymyxins, where polypropylene plates (Griener Bio-One Ltd, Stonehouse, UK) were utilised. Bacterial growth in the presence of biocides was measured by taking an OD_600_ reading every hour for 20 hours using a FLUOstar Omega plate reader (BMG Labtech GMBH, Ortenberg, Germany). For MBC testing, 10 µl of suspension was removed from each well of the MIC microtiter plate where no bacterial growth was observed, along with the two wells immediately below the MIC where growth was observed. These were spotted on TSA plates and incubated at 37 °C for 24 h. The MBC was defined as the lowest concentration of antibiotic/disinfectant at which no bacterial growth was observed in three replicate experiments.

### Whole genome sequencing and Real Time-PCR

This was carried out as previously described^11^ and PHE Galaxy was used to analyse genetic changes^37^. Quantitative Real Time PCR was carried out and analysed as previously described using primers already described^11^. Exposure to chlorhexidine or octenidine was for 30 mins.

### Cloning *smvA(R)* fragments into *E. coli*

*K. pneumoniae* and *S. enterica* serovar Typhimurium *smvA* and *smvAR* fragments were amplified from strains MGH 78578 and SL1344 respectively using primers described in Supplementary Table 4. Fragments were subsequently cloned into pCR 2.1 TOPO vector (Invitrogen) according to manufacturer’s instructions. Plasmids were then transformed into *E. coli* TOP10 cells and positive colonies selected for on TSA plates containing kanamycin (30 mg/L). Plasmid transformation was confirmed by PCR using standard M13 forward and reverse primers. A selection of plasmids were purified and the *smvA(R)* fragment sequence confirmed by Sanger sequencing.

### Homology modelling and generation of the SmvA Structures

The interaction of the wild-type SmvA efflux pump and its A363V and Y391N mutant forms in complex with different ligands was explored using computational methods including molecular docking, molecular dynamics (MD) simulation, molecular mechanics, Poisson-Boltzmann surface area/molecular mechanics and generalised Born surface area (MM-PBSA/MM-GBSA) calculation. Homology modelling was utilised for the generation of the structural model of the transporter in PDB format. The I-TASSER webserver^38–40^ was used for the homology modelling of SmvA structural model from *K. pneumoniae*, using the FASTA formatted target sequence with UniProt entry number of A6T9N7. C-score is a confidence score for estimating the quality of predicted models by I-TASSER, and a C-score of −1.08 was obtained for this model which is well within the acceptable range [−5, 2]. The generated model was without any gap, and all the segments were solved. Accelrys Discovery Studio 2017 was used to add probable missing side chains in the generated model. The mutant forms of SmvA were generated as A363V and Y391N individually, by the PyMOL program. The structures were minimised by the AMBER package program^41,42^ before carrying out the molecular docking and running MD simulations. Additional computational modelling methods are listed in supplementary methods.

### Statistical analysis

All bacterial growth curves were analysed by calculating the time taken to reach half the maximal OD and then comparing these values with relevant controls using a Students Unpaired T-test. Real Time PCR data was analysed for significance using the Students Unpaired T-test. For significance *P* values < 0.0001 ****; 0.001–0.0001 ***; 0.01–0.001 **; 0.05–0.01 *; ≥0.05 non-significant were used.

## Supplementary information

Supplementary Data
