# Supplementary material for: SmvA is an important efflux pump for cationic biocides in Klebsiella pneumoniae and other Enterobacteriaceae
Source: Sci Rep. 2019 Feb 4;9:1344. doi: 10.1038/s41598-018-37730-0 (PMC6362122; doi:10.1038/s41598-018-37730-0)
Supplement: Supplementary file 1 — Supplementary Data [file 41598_2018_37730_MOESM1_ESM.pdf]

Supplementary Material for:

SmvA is an important efflux pump for cationic biocides in *Klebsiella pneumoniae* and other Enterobacteriaceae.

Matthew E Wand, Shirin Jamshidi, Lucy J Bock, Khondaker Miraz Rahman, and J Mark Sutton

| Clone | Phenotype | Position of transposon in gene | Direction in genome |
| --- | --- | --- | --- |
| KP05925 | Δ*smvA* | 172 (1485) | Forward |
| KP05926 | Δ*smvR1* | 532 (576) | Forward |
| KP05927 | Δ*smvR2* | 146 (576) | Forward |
| KP05928 | Δ*smvR3* | 512 (576) | Reverse |

**Supplementary Table 1 -** Characteristics of transposon mutants. The approximate position of the transposon insertion is given in bp relative to the full length gene (Number in brackets).

| | CIP | NAL | LVX | NOR | MXF | ERY | RIF | DOX |
|---|---|---|---|---|---|---|---|---|
| MKP103 | >512 | >512 | 512 | >512 | 256-512 | >512 | 32 | 64 |
| *ΔsmvA* | >512 | >512 | 512 | >512 | 256 | >512 | 32 | 64 |
| *ΔsmvR1* | >512 | >512 | 512 | >512 | 256 | >512 | 32 | 64 |
| *ΔsmvR2* | >512 | >512 | 512 | >512 | 256 | >512 | 32 | 64 |
| *ΔsmvR3* | >512 | >512 | 512 | >512 | 256 | >512 | 32 | 64 |

| | STR | GEN | TOB | CST | AZM | TGC | CAZ | CTX |
|---|---|---|---|---|---|---|---|---|
| MKP103 | 128 | 8 | 128 | >64 | >256 | 2-4 | 2 | 2-4 |
| *ΔsmvA* | 128 | 8-16 | 128 | >64 | >256 | 4 | 2 | 2 |
| *ΔsmvR1* | 128 | 8 | 128 | >64 | >256 | 2-4 | 2 | 2 |
| *ΔsmvR2* | 128 | 8 | 128 | >64 | >256 | 2-4 | 2 | 2 |
| *ΔsmvR3* | 128 | 8 | 128 | >64 | >256 | 2-4 | 2 | 2 |

**Supplementary Table 2 -** MICs of transposon mutants to various antibiotics. All values are given in mg/L. For abbreviations CIP, ciprofloxacin; NAL, nalidixic acid; LVX, levofloxacin; NOR, norfloxacin; MXF, moxifloxacin; ERY, erythromycin; RIF, rifampicin; DOX, doxycycline; STR, streptomycin; GEN, gentamicin; TOB, tobramycin; CST, colistin; AZM, azithromycin; TGC, tigecycline; CAZ, ceftazidime; CTX, cefotaxime.

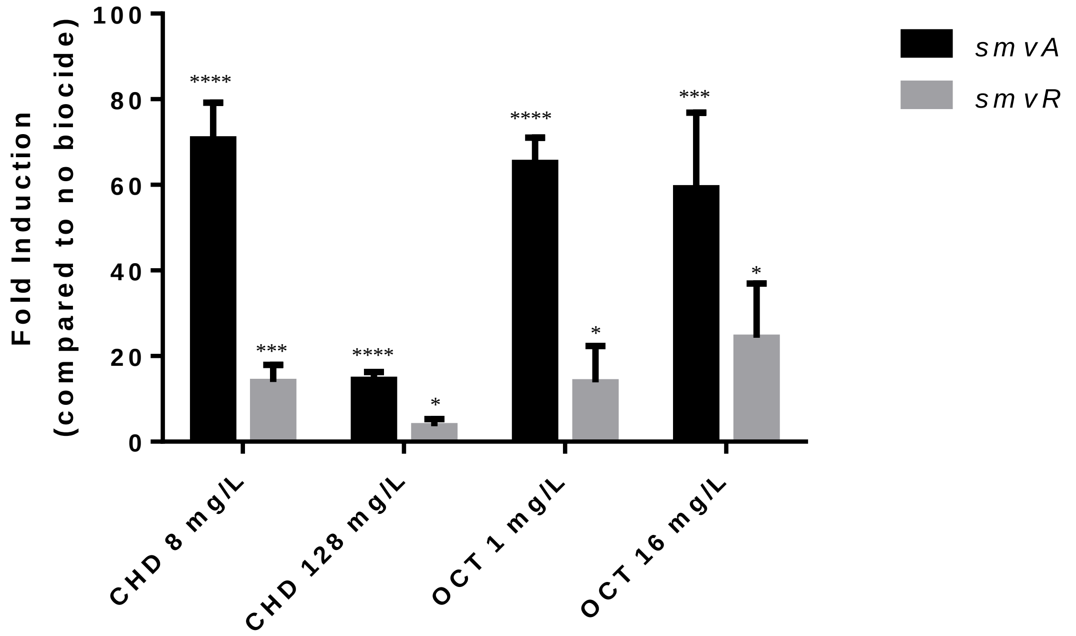


**Supplementary Figure S1** – Fold induction of *smvA* and *smvR* in *K. pneumoniae* strain MGH 78578 following challenge with chlorhexidine (CHD) or octenidine (OCT) at the concentrations indicated. All experiments were performed in triplicate and the results are shown as the mean fold induction in comparison to challenge with no biocide. Error bars represent the standard deviation. Statistical significance is shown.

| Isolate | MIC (mg/L) | Isolate | MIC (mg/L) |
|---|---|---|---|
| NCTC 10418 | 0.5-1 | LEC014 | 1 |
| NCTC 12923 | 0.5-1 | LEC016 | 1 |
| LEC001 | 0.5-1 | LEC053 | 0.5-1 |
| LEC006 | 4-8 | LEC070 | 0.5-1 |
| LEC064 | 0.5-1 | LEC085 | 0.5-1 |
| LEC067 | 0.5-1 | LEC094 | 1 |
| LEC080 | 0.5 | CFI_017_VIM4 | 0.5-1 |
| LEC107 | 1-2 | CFI_140_NDM-1 | 0.5-1 |
| LEC095 | 0.5-1 | CFI_160_KPC2 | 0.5 |
| LEC007 | 0.5 | CFI_162_OXA48 | 0.5-1 |
| LEC008 | 0.5 | MG1655 | 0.5 |
| LEC013 | 0.5 | DH5 | 0.5 |

**Supplementary Table 3** – MIC values for chlorhexidine for a range of *E. coli* isolates. Values shown are the range from 3 independent repeats.

```
                 ◄ smvA                  DYAD P1                DYAD P2          DYAD  P3                                  smvR ►
M585            CCATTGCCGTGACATAGCTTGTACCTCAATCAATATT-GAATATTGGACACACGTCCAGGATGGCGATCCTGCCGGATTGTTGAACACATGTCCAACCTTTTGTTACACTCGGCCAAAGCCAGATGACAGCGGGGTTCT - ST3
M586            CCATTGCCGTGACATAGCTTGTACCTCAATCAATATT-GAATATTGGACACACGTCCAGGATGGCGATCCTGCCGGATTGTTGAACACATGTCCAACCTTTTGTTACACTCGGCCAAAGCCAGATGACAGCGGGGTTCT - ST3
NCTC 9633       CCATTGCCGTGACATAGCTTGTACCTCAATCAATATT-GAATATTGGACACACGTCCAGGATGGCGATCCTGCCGGATTGTTGAACACATGTCCAACCTTTTGTTACACTCGGCCAAAGCCAGATGACAGCGGGGTTCT - ST3
BS102           CCATTGCCGTGACATAGCTTGTACCTCAATCAATATT-GAATATTGGACACACGTCCAGGATGGCGATCCTGCCGGATTGTTGAACACATGTCCAACTTTTTGTTACACTCGGCCAAAGCCAGATGACAGCGGGGTTCT - ST10
2619            CCATTGCCGTGACATAGCTTGTACCTCAATCAATATT-GAATATTGGACACACGTCCAGGATGGCGATCCTGCCGGATTGTTGAACACATGTCCAACTTTTTGTTACACTCGGCCAAAGCCAGATGACAGTGGGGTTCT - ST11
2274            CCATTGCCGTGACATAGCTTGTACCTCAATCAATATT-GAATATTGGACACACGTCCAGGATGGCGATCCTGCCGGATTGTTGAACACATGTCCAACTTTTTGTTACACTCGGCCAAAGCCAGATGACAGTGGGGTTCT - ST11
2609            CCATTGCCGTGACATAGCTTGTACCTCAATCAATATT-GAATATTGGACACACGTCCAGGATGGCGATCCTGCCGGATTGTTGAACACATGTCCAACTTTTTGTTACACTCGGCCAAAGCCAGATGACAGTGGGGTTCT - ST11
2660            CCATTGCCGTGACATAGCTTGTACCTCAATCAATATT-GAATATTGGACACACGTCCAGGATGGCGATCCTGCCGGATTGTTGAACACATGTCCAACTTTTTGTTACACTCGGCCAAAGCCAGATGACAGTGGGGTTCT - ST11
2344            CCATTGCCGTGACATAGCTTGTACCTCAATCAATATT-GAATATTGGACACACGTCCAGGATGGCGATCCTGCCGGATTGTTGAACACATGTCCAACTTTTTGTTACACTCGGCCAAAGCCAGATGACAGTGGGGTTCT - ST11
2638            CCATTGCCGTGACATAGCTTGTACCTCAATCAATATT-GAATATTGGACACACGTCCAGGATGGCGATCCTGCCGGATTGTTGAACACATGTCCAACTTTTTGTTACACTCGGCCAAAGCCAGATGACAGTGGGGTTCT - ST11
2627            CCATTGCCGTGACATAGCTTGTACCTCAATCAATATT-GAATATTGGACACACGTCCAGGATGGCGATCCTGCCGGATTGTTGAACACATGTCCAACTTTTTGTTACACTCGGCCAAAGCCAGATGACAGTGGGGTTCT - ST11
CFI_127_NDM-1   CCATTGCCGTGACATAGCTTGTACCTCAATCAATATT-GAATATTGGACACACGTCCAGGATGGCGATCCTGCCGGATTGTTGAACACATGTCCAACTTTTTGTTACACTCGGCCAAAGCCAGATGACAGTGGGGTTCT - ST11
CFI_108_OXA-48  CCATTGCCGTGACATAGCTTGTACCTCAATCAATATT-GAATATTGGACACACGTCCAGGATGGCGATCCTGCCGGATTGTTGAACACATGTCCAACTTTTTGTTACACTCGGCCAAAGCCAGATGACAGTGGGGTTCT - ST11
BS27            CCATTGCCGTGACATAGCTTGTACCTCAATCAATATT-GAATATTGGACACACGTCCAGGATGGCGATCCTGCCGGATTGTTGAACACATGTCCAACTTTTTGTTACACTCGGCCAAAGCCAGATGACAGTGGGGTTCT - ST11
NCTC 13443      CCATTGCCGTGACATAGCTTGTACCTCAATCAATATT-GAATATTGGACACACGTCCAGGATGGCGATCCTGCCGGATTGTTGAACACATGTCCAACTTTTTGTTACACTCGGCCAAAGCCAGATGACAGCGGGGTTCT - ST14
KPUK04a         CCATTGCCGTGACATAGCTTGTACCTCAATCAATATT-GAATATTGGACACACGTCCAGGATGGCGATCCTGCCGGATTGTTGAACACATGTCCAACTTTTTGTTACACTCGGCCAAAGCCAGATGACAGCGGGGTTCT - ST14
KPUK04b         CCATTGCCGTGACATAGCTTGTACCTCAATCAATATT-GAATATTGGACACACGTCCAGGATGGCGATCCTGCCGGATTGTTGAACACATGTCCAACTTTTTGTTACACTCGGCCAAAGCCAGATGACAGCGGGGTTCT - ST14
CFI_112_NDM1    CCATTGCCGTGACATAGCTTGTACCTCAATCAATATT-GAATATTGGACACACGTCCAGGATGGCGATCCTGCCGGATTGTTGAACACATGTCCAACTTTTTGTTACACTCGGCCAAAGCCAGATGACAGCGGGGTTCT - ST14
CFI_134_NDM1    CCATTGCCGTGACATAGCTTGTACCTCAATCAATATT-GAATATTGGACACACGTCCAGGATGGCGATCCTGCCGGATTGTTGAACACATGTCCAACTTTTTGTTACACTCGGCCAAAGCCAGATGACAGCGGGGTTCT - ST15
18              CCATTGCCGTGACATAGCTTGTACCTCAATCAATATT-GAATATTGGACACACGTCCAGGATGGCGATCCTGCCGGATTGTTGAACACATGTCCAACTTTTTGTTACACTCGGCCAAAGCCAGATGACAGCGGGGTTCT - ST15
CFI_145_NDM1    CCATTGCCGTGACATAGCTTGTACCTCAATCAATATT-GAATATTGGACACACGTCCAGGATGGCGATCCTGCCGGATTGTTGAACACATGTCCAACTTTTTGTTACACTCGGCCAAAGCCAGATGACAGCGGGGTTCT - ST15
KPUK01          CCATTGCCGTGACATAGCTTGTACCTCAATCAATATT-GAATATTGGACACACGTCCAGGATGGCGATCCTGCCGGATTGTTGAACACATGTCCAACTTTTTGTTACACTCGGCCAAAGCCAGATGACAGCGGGGTTCT - ST15
M1              CCATTGCCGTGACATAGCTTGTACCTCAATCAATATT-GAATATTGGACACACGTCCAGGATGGCGATCCTGCCGGATTGTTGAACACATGTCCAACTTTTTGTTACACTCGGCCAAAGCCAGATGACAGCGGGGTTCT - ST22
M109            CCATTGCCGTGACATAGCTTGTACCTCAATCAATATT-GAATATTGGACACACGTCCAGGATGGCGATCCTGCCGGATTGTTGAACACATGTCCAACTTTTTGTTACACTCGGCCAAAGCCAGATGACAGTGGGGTTCT - ST23
TW3             CCATTGCCGTGACATAGCTTGTACCTCAATCAATATT-GAATATTGGACACACGTCCAGGATGGCGATCCTGCCGGATTGTTGAACACATGTCCAACTTTTTGTTACACTCGGCCAAAGCCAGATGACAGTGGGGTTCT - ST23
M6              CCATTGCCGTGACATAGCTTGTACCTCAATCAATATT-GAATATTGGACACACGTCCAGGATGGCGATCCTGCCGGATTGTTGAACACATGTCCAACTTTTTGTTACACTCGGCCAAAGCCAGATGACAGTGGGGTTCT - ST23
4               CCATTGCCGTGACATAGCTTGTACCTCAATCAATATT-GAATATTGGACACACGTCCAGGATGGCGATCCTGCCGGATTGTTGAACACATGTCCAACTATTTGTTACACTCGGCCAAAGCCAGATGACAGCGGGGTTCT - ST25
5               CCATTGCCGTGACATAGCTTGTACCTCAATCAATATT-GAATATTGGACACACGTCCAGGATGGCGATCCTGCCGGATTGTTGAACACATGTCCAACTATTTGTTACACTCGGCCAAAGCCAGATGACAGCGGGGTTCT - ST25
25              CCATTGCCGTGACATAGCTTGTACCTCAATCAATATT-GAATATTGGACACACGTCCAGGATGGCGATCCTGCCGGATTGTTGAACACATGTCCAACTTTTTGTTACACTCGGCCAAAGCCAGATGACAGCGGGGTTCT - ST34
12              CCATTGCCGTGACATAGCTTGTACCTCAATCAATATT-GAATATTGGACGCACGTCCAGGATGGCGATCCTGCCGGATTGTTGAACACATGTCCAACTTTTTGTTACACTCGGCCAAAGCCAGATGACAGCGGGGTTCT - ST35
M295            CCATTGCCGTGACATAGCTTGTACCTCAATCAATATT-GAATATTGGACGCACGTCCAGGATGGCGATCCTGCCGGATTGTTGAACACATGTCCAACTTTTTGTTACACTCGGCCAAAGCCAGATGACAGCGGGGTTCT - ST35
M296            CCATTGCCGTGACATAGCTTGTACCTCAATCAATATT-GAATATTGGACACACGTCCAGGATGGCGATCCTGCCGGATTGTTGAACACATGTCCAACTTTTTGTTACACTCGGCCAAAGCCAGATGACAGTGGGGTTCT - ST36
MGH 78578       CCATTGCCGTGACATAGCTTGTACCTCAATCAATATT-GAATATTGGACACACGTCCAGGATGGCGATCCTGCCGGATTGTTGAACACATGTCCAACTTTTTGTTACACTCGGCCAAAGCCAGATGACAGCGGGGTTCT - ST38
17              CCATTGCCGTGACATAGCTTGTACCTCAATCAATATT-GAATATTGGACACACGTCCAGGATGGCGATCCTGCCGGATTGTTGAACACATGTCCAACTTTTTGTTACACTCGGCCAAAGCCAGATGACAGCGGGGTTCT - ST45
19              CCATTGCCGTGACATAGCTTGTACCTCAATCAATATT-GAATATTGGACACACGTCCAGGATGGCGATCCTGCCGGATTGTTGAACACATGTCCAACTTTTTGTTACACTCGGCCAAAGCCAGATGACAGCGGGGTTTT - ST48
NCTC 13439      CCATTGCCGTGACATAGCTTGTACCTCAATCAATATT-GAATATTGGACACACGTCCAGGATGGCGATCCTGCCGGATTGTTGAACACATGTCCAACTTTTTGTTACACTCGGCCAAAGCCAGATGACAGCGGGGTTCT - ST54
KPUK03          CCATTGCCGTGACATAGCTTGTACCTCAATCAATATT-GAATATTGGACACACGTCCAGGATGGCGATCCTGCCGGATTGTTGAACACATGTCCAACTTTTTGTTACACTCGGCCAAAGCCAGATGACAGCGGGGTTCT - ST65
M44             CCATTGCCGTGACATAGCTTGTACCTCAATCAATATT-GAATATTGGACACACGTCCAGGATGGCGATCCTGCCGGATTGTTGAACACATGTCCAACTTTTTGTTACACTCGGCCAAAGCCAGATGACAGCGGGGTTCT - ST82
M45             CCATTGCCGTGACATAGCTTGTACCTCAATCAATATT-GAATATTGGACACACGTCCAGGATGGCGATCCTGCCGGATTGTTGAACACATGTCCAACTTTTTGTTACACTCGGCCAAAGCCAGATGACAGCGGGGTTCT - ST82
M69             CCATTGCCGTGACATAGCTTGTACCTCAATCAATATT-GAATATTGGACACACGTCCAGGATGGCGATCCTGCCGGATTGTTGAACACATGTCCAACTTTTTGTTACACTCGGCCAAAGCCAGATGACAGCGGGGTTCT - ST82
M175A           CCATTGCCGTGACATAGCTTGTACCTCAATCAATATT-GAATATTGGACACACGTCCAGGATGGCGATCCTGCCGGATTGTTGAACACATGTCCAACTTTTTGTTACACTCGGCCAAAGCCAGATGACAGCGGGGTTCT - ST82
M342            CCATTGCCGTGACATAGCTTGTACCTCAATCAATATT-GAATATTGGACACACGTCCAGGATGGCGATCCTGCCGGATTGTTGAACACATGTCCAACTTTTTGTTACACTCGGCCAAAGCCAGATGACAGCGGGGTTCT - ST82
M343            CCATTGCCGTGACATAGCTTGTACCTCAATCAATATT-GAATATTGGACACACGTCCAGGATGGCGATCCTGCCGGATTGTTGAACACATGTCCAACTTTTTGTTACACTCGGCCAAAGCCAGATGACAGCGGGGTTCT - ST82
M345            CCATTGCCGTGACATAGCTTGTACCTCAATCAATATT-GAATATTGGACACACGTCCAGGATGGCGATCCTGCCGGATTGTTGAACACATGTCCAACTTTTTGTTACACTCGGCCAAAGCCAGATGACAGCGGGGTTCT - ST82
M371            CCATTGCCGTGACATAGCTTGTACCTCAATCAATATT-GAATATTGGACACACGTCCAGGATGGCGATCCTGCCGGATTGTTGAACACATGTCCAACTTTTTGTTACACTCGGCCAAAGCCAGATGACAGCGGGGTTCT - ST82
M372            CCATTGCCGTGACATAGCTTGTACCTCAATCAATATT-GAATATTGGACACACGTCCAGGATGGCGATCCTGCCGGATTGTTGAACACATGTCCAACTTTTTGTTACACTCGGCCAAAGCCAGATGACAGCGGGGTTCT - ST82
M414            CCATTGCCGTGACATAGCTTGTACCTCAATCAATATT-GAATATTGGACACACGTCCAGGATGGCGATCCTGCCGGATTGTTGAACACATGTCCAACTTTTTGTTACACTCGGCCAAAGCCAGATGACAGCGGGGTTCT - ST82
M583            CCATTGCCGTGACATAGCTTGTACCTCAATCAATATT-GAATATTGGACACACGTCCAGGATGGCGATCCTGCCGGATTGTTGAACACATGTCCAACTTTTTGTTACACTCGGCCAAAGCCAGATGACAGCGGGGTTCT - ST82
M584            CCATTGCCGTGACATAGCTTGTACCTCAATCAATATT-GAATATTGGACACACGTCCAGGATGGCGATCCTGCCGGATTGTTGAACACATGTCCAACTTTTTGTTACACTCGGCCAAAGCCAGATGACAGCGGGGTTCT - ST82
M587            CCATTGCCGTGACATAGCTTGTACCTCAATCAATATT-GAATATTGGACACACGTCCAGGATGGCGATCCTGCCGGATTGTTGAACACATGTCCAACTTTTTGTTACACTCGGCCAAAGCCAGATGACAGCGGGGTTCT - ST82
M588            CCATTGCCGTGACATAGCTTGTACCTCAATCAATATT-GAATATTGGACACACGTCCAGGATGGCGATCCTGCCGGATTGTTGAACACATGTCCAACTTTTTGTTACACTCGGCCAAAGCCAGATGACAGCGGGGTTCT - ST82
M636            CCATTGCCGTGACATAGCTTGTACCTCAATCAATATT-GAATATTGGACACACGTCCAGGATGGCGATCCTGCCGGATTGTTGAACACATGTCCAACTTTTTGTTACACTCGGCCAAAGCCAGATGACAGCGGGGTTCT - ST82
16              CCATTGCCGTGACATAGCTTGTACCTCAATCAATATT-GAATATTGGACACACGTCCAGGATGGCGATCCTGCCGGATTGTTGAACACATGTCCAACTTTTTGTTACACTCGGCCAAAGCCAGATGACAGCGGGGTTCT - ST101
CFI_110_OXA232  CCATTGCCGTGACATAGCTTGTACCTCAATCAATATT-GAATATTGGACACACGTCCAGGATGGCGATCCTGCCGGATTGTTGAACACATGTCCAACTTTTTGTTACACTCGGCCAAAGCCAGATGACAGCGGGGTTCT - ST101
CFI_119_OXA48   CCATTGCCGTGACATAGCTTGTACCTCAATCAATATT-GAATATTGGACACACGTCCAGGATGGCGATCCTGCCGGATTGTTGAACACATGTCCAACTTTTTGTTACACTCGGCCAAAGCCAGATGACAGCGGGGTTCT - ST101
CFI_118_NDM1    CCATTGCCGTGACATAGCTTGTACCTCAATCAATATT-GAATATTGGACACACGTCCAGGATGGCGATCCTGCCGGATTGTTGAACACATGTCCAACTTTTTGTTACACTCGGCCAAAGCCAGATGACAGCGGGGTTCT - ST101
CFI_147_OXA48   CCATTGCCGTGACATAGCTTGTACCTCAATCAATATT-GAATATTGGACACACGTCCAGGATGGCGATCCTGCCGGATTGTTGAACACATGTCCAACTTTTTGTTACACTCGGCCAAAGCCAGATGACAGCGGGGTTCT - ST101
CFI_151_OXA48   CCATTGCCGTGACATAGCTTGTACCTCAATCAATATT-GAATATTGGACACACGTCCAGGATGGCGATCCTGCCGGATTGTTGAACACATGTCCAACTTTTTGTTACACTCGGCCAAAGCCAGATGACAGCGGGGTTCT - ST101
CFI_145_KPC2    CCATTGCCGTGACATAGCTTGTACCTCAATG----TTAGAA-CTTGGACACACGTCCAGGATGGCGATCCTGCCGGATTGTTGAACGCATGTCCAACTTTTTGTTACACTCGGCCAAAGCCAGATATCAGCGGGGTTCT - ST138
342             CCATTGCCGTGACATAGCTTGTACCTCAATG----TTGGAA-ATTGGACACACGTCCAGGATGGCGATCCTGCCGGAATGTTGAACGCATGTCCAACTTTTTGTTACACTCGGCCAAAGCCAGATGACAGCGGGGTTCT - ST146
Leeds19         CCATTGCCGTGACATAGCTTGTACCTCAATG----TTGGAA-ATTGGACACACGTCCAGGATGGCGATCCTGCCGGATTGTTGAACGCATGTCCAACTTTTTGTTACACTCGGCCAAAGCCAGATGACAGCGGGGTTCT - ST197
Leeds1          CCATTGCCGTGACATAGCTTGTACCTCAATG----TTGGAA-ATTGGACACACGTCCAGGATGGCGATCCTGCCGGATTGTTGAACGCATGTCCAACTTTTTGTTACACTCGGCCAAAGCCAGATGACAGCGGGGTTCT - ST250
NCTC 13438      CCATTGCCGTGACATAGCTTGTACCTCAATCAATATT-GAATATTGGACACACGTCCAGGATGGCGATCCTGCCGGATTGTTGAACACATGTCCAACTTTTTGTTACACTCGGCCAAAGCCAGATGACAGCGGGGTTTT - ST258
51851           CCATTGCCGTGACATAGCTTGTACCTCAATCAATATT-GAATATTGGACACACGTCCAGGATGGCGATCCTGCCGGATTGTTGAACACATGTCCAACTTTTTGTTACACTCGGCCAAAGCCAGATGACAGCGGGGTTTT - ST258
```

```
46704           CCATTGCCGTGACATAGCTTGTACCTCAATCAATATT-GAATATTGGACACACGTCCAGGATGGCGATCCTGCCGGATTGTTGAACACATGTCCAACTTTTTGTTACACTCGGCCAAAGCCAGATGACAGCGGGGTTTT - ST258
45701           CCATTGCCGTGACATAGCTTGTACCTCAATCAATATT-GAATATTGGACACACGTCCAGGATGGCGATCCTGCCGGATTGTTGAACACATGTCCAACTTTTTGTTACACTCGGCCAAAGCCAGATGACAGCGGGGTTTT - ST258
49856           CCATTGCCGTGACATAGCTTGTACCTCAATCAATATT-GAATATTGGACACACGTCCAGGATGGCGATCCTGCCGGATTGTTGAACACATGTCCAACTTTTTGTTACACTCGGCCAAAGCCAGATGACAGCGGGGTTTT - ST258
CFI_141_KPC3    CCATTGCCGTGACATAGCTTGTACCTCAATCAATATT-GAATATTGGACACACGTCCAGGATGGCGATCCTGCCGGATTGTTGAACACATGTCCAACTTTTTGTTACACTCGGCCAAAGCCAGATGACAGCGGGGTTTT - ST258
CFI_140_KPC3    CCATTGCCGTGACATAGCTTGTACCTCAATCAATATT-GAATATTGGACACACGTCCAGGATGGCGATCCTGCCGGATTGTTGAACACATGTCCAACTTTTTGTTACACTCGGCCAAAGCCAGATGACAGCGGGGTTTT - ST258
MKP103          CCATTGCCGTGACATAGCTTGTACCTCAATCAATATT-GAATATTGGACACACGTCCAGGATGGCGATCCTGCCGGATTGTTGAACACATGTCCAACTTTTTGTTACACTCGGCCAAAGCCAGATGACAGCGGGGTTTT - ST258
CFI_131_KPC2    CCATTGCCGTGACATAGCTTGTACCTCAATCAATATT-GAATATTGGACACACGTCCAGGATGGCGATCCTGCCGGATTGTTGAACACATGTCCAACTTTTTGTTACACTCGGCCAAAGCCAGATGACAGCGGGGTTTT - ST258
CFI_147_KPC2    CCATTGCCGTGACATAGCTTGTACCTCAATCAATATT-GAATATTGGACACACGTCCAGGATGGCGATCCTGCCGGATTGTTGAACACATGTCCAACTTTTTGTTACACTCGGCCAAAGCCAGATGACAGCGGGGTTTT - ST258
6               CCATTGCCGTGACATAGCTTGTACCTCAATCAATATT-GAATATTGGACACACGTCCAGGATGGCGATCCTGCCGGATTGTTGAACACATGTCCAACTTTTTGTTACACTCGGCCAAAGCCAGATGACAGTGGGGTTCT - ST268
M3              CCATTGCCGTGACATAGCTTGTACCTCAATCAATATT-GAATATTGGACACACGTCCAGGATGGCGATCCTGCCGGATTGTTGAACACATGTCCAACTTTTTGTTACACTCGGCCAAAGCCAGATGACAGCGGGGTTCT - ST336
20              CCATTGCCGTGACATAGCTTGTACCTCAATCAATATT-GAATATTGGACACACGTCCAGGATGGCGATCCTGCCGGATTGTTGAACACATGTCCAACTTTTTGTTACACTCGGCCAAAGCCAGATGACAGCGGGGTTCT - ST336
5595            CCATTGCCGTGACATAGCTTGTACCTCAATG----TTGGAA-ATTGGACACACGTCCAGGATGGCGATCCTGCCGGATTGTTGAACGCATGTCCAATTTTTTGTTACACTCGGCCAAAGTCAGATGACAGCGGGGTTCT - ST347
15              CCATTGCCGTGACATAGCTTGTACCTCAATCAATATT-GAATATTGGACACACGTCCAGGATGGCGATCCTGCCGGATTGTTGAACACATGTCCAACTTTTTGTTACACTCGGCCAAAGCCAGATGACAGCGGGGTTCT - ST353
NCTC 13368      CCATTGCCGTGACATAGCTTGTACCTCAATG----TTAGAA-CTTGGACACACGTCCAGGATGGCGATCCTGCCGGATTGTTGAACGCATGTCCAACTTTTTGTTACACTCGGCCAAAGCCAGATATCAGCGGGGTTCT - ST489
9               CCATTGCCGTGACATAGCTTGTACCTCAATCAATATT-GAATATTGGACACACGTCCAGGATGGCGATCCTGCCGGATTGTTGAACACATGTCCAACTTTTTGTTACACTCGGCCAAAGCCAGATGACAGCGGGGTTCT - ST711
BS26            CCATTGCCGTGACATAGCTTGTACCTCAATCAATATT-GAATATTGGACACACGTCCAGGATGGCGATCCTGCCGGATTGTTGAACACATGTCCAACTTTTTGTTACACTCGGCCAAAGCCAGATGACAGTGGGGTTCT - ST922
BS11            CCATTGCCGTGACATAGCTTGTACCTCAATCAATATT-GAATATTGGACACACGTCCAGGATGGCGATCCTGCCGGATTGTTGAACACATGTCCAACTTTTTGTTACACTCGGCCAAAGCCAGATGACAGTGGGGTTCT - ST922
CFI_129_OXA48   CCATTGCCGTGACATAGCTTGTACCTCAATG----TTGGAA-ATTGGACACACGTCCAGGATGGCGATCCTGCCGGATTGTTGAACGCATGTCCAACTTTTTGTTACACTCGGCCAAAGCCAGATGACAGCGGGGTTCT - ST1562
11              CCATTGCCGTGACATAGCTTGTACCTCAATG----TTGGAA-ATTGGACACACGTCCAGGATGGCGATCCTGCCGGATTGTTGAACGCATGTCCAACTTTTTGTTACACTCGGCCAAAGCCAGATGACAGCGGGGTTCT - ST1688
M42             CCATTGCCGTGACATAGCTTGTACCTCAATCAATATT-GAATATTGGACACACGTCCAGGATGGCGATCCTGCCGGATTGTTGAACACATGTCCAACTTTTTGTTACACTCGGCCAAAGCCAGATGACAGCGGGGTTCT - STND
M70             CCATTGCCGTGACATAGCTTGTACCTCAATCAATATT-GAATATTGGACACACGTCCAGGATGGCGATCCTGCCGGATTGTTGAACACATGTCCAACTTTTTGTTACACTCGGCCAAAGCCAGATGACAGCGGGGTTCT - STND
M124            CCATTGCCGTGACATAGCTTGTACCTCAATCAATATT-GAATATTGGACACACGTCCAGGATGGCGATCCTGCCGGATTGTTGAACACATGTCCAACTTTTTGTTACACTCGGCCAAAGCCAGATGACAGCGGGGTTCT - STND
M176            CCATTGCCGTGACATAGCTTGTACCTCAATCAATATT-GAATATTGGACACACGTCCAGGATGGCGATCCTGCCGGATTGTTGAACACATGTCCAACTTTTTGTTACACTCGGCCAAAGCCAGATGACAGCGGGGTTCT - STND
M426            CCATTGCCGTGACATAGCTTGTACCTCAATCAATATT-GAATATTGGACACACGTCCAGGATGGCGATCCTGCCGGATTGTTGAACACATGTCCAACTTTTTGTTACACTCGGCCAAAGCCAGATGACAGTGGGGTTCT - STND
M665            CCATTGCCGTGACATAGCTTGTACCTCAATCAATATT-GAATATTGGACACACGTCCAGGATGGCGATCCTGCCGGATTGTTGAACACATGTCCAACTTTTTGTTACACTCGGCCAAAGCCAGATGACAGCGGGGTTCT - STND
5596            CCATTGCCGTGACATAGCTTGTACCTCAATG----TTGGAA-ATTGGACACACGTCCAGGATGGCGATCCTGCCGGAATGTTGAACGCATGTCCAACTTTTTGTTACACTCGGCCAAAGCCAGATGACAGCGGGGTTCT - STND
CFI_130_NDM-1   CCATTGCCGTGACATAGCTTGTACCTCAATCAATATT-GAATATTGGACACACGTCCAGGATGGCGATCCTGCCGGATTGTTGAACACATGTCCAACTTTTTGTTACACTCGGCCAAAGCCAGATGACAGCGGGGTTCT - STND
```

**Supplementary Figure S2** – Alignment of *smvAR* intergenic region in 91 *K. pneumoniae* strains. The DYAD motifs are coloured and strains from the same sequence type are indicated. Base pair changes differing from the consensus sequence are indicated in red. DYAD region P2 is absolutely conserved and DYAD region P3 which is the region where mutations following chlorhexidine adaptation were found is mostly conserved. Several strains have deleted part of DYAD region P1 which may indicate that this is not important for SmvR binding. The sequence type (ST) is indicated for all strains. ND means not determined.

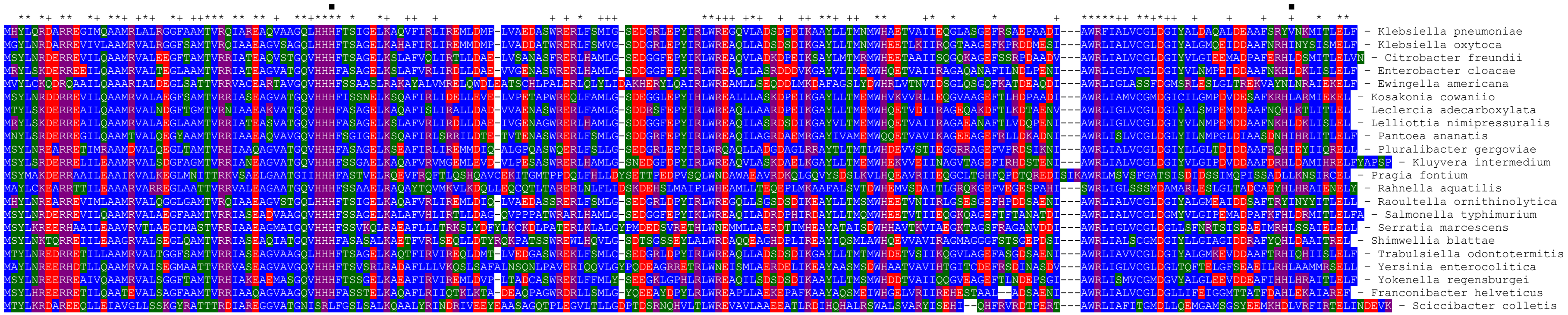


**Supplementary Fig. S3** – Alignment of SmvR sequences from members of the Enterobacteriaceae. Amino acids are classified according to their sidechains (Non-polar – Blue; Uncharged Polar – Green; Polar Acidic –Red; Polar Basic –Purple). Residues of high homology (>90% *), areas of high identity (>90% +) are shown above the sequences. Amino acids which have been changed following chlorhexidine adaptation are also shown above the sequences (■). Sequences are based on the consensus of 20 sequences where possible. Other members of the Enterobacteriaceae e.g. *Proteus*, *Providencia*, *Chronobacter* and *Hafnia* sp. also have predicted homologues to SmvR but their sequences are very diverse from the *K. pneumoniae* SmvR. All protein sequences were aligned using Clustal X and MegAlign Pro 14.

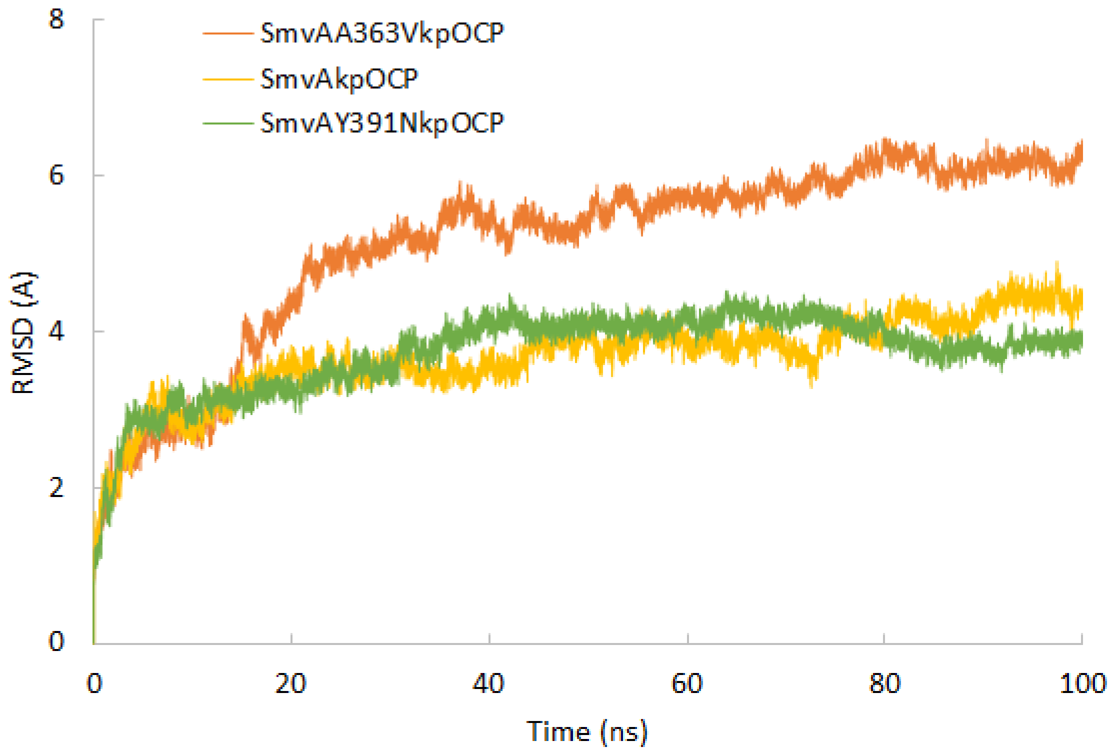


**Supplementary Fig. S4** - Time dependence of RMSD (Å) for the backbone atoms relative to the starting structure during 100 ns MD simulations of the SmvA-Octenidine complexes. RMSD curves show that all simulations have reached steady state after ~ 35 ns indicated by the relatively stable RMSD values from 35 ns to the end of the simulations.

| Primer Name | Primer Sequence | Use |
|---|---|---|
| KP SmvA TOPO Rev | CAGCTATAGCGCCGACTGCGG | Used to amplify *K. pneumoniae smvA* or *smvAR* |
| KP SmvA Prom TOPO For | CTGGGCTTCGCGGGCGATCTG | Used to amplify *K. pneumoniae smvA* |
| KP SmvR TOPO Rev | TGCCTAAAACAACTCCAGCGT | Used to amplify *K. pneumoniae smvAR* |
| SalTy SmvA R ClaI | TAGCATGCATTTATCGGCGTTGGGCTTTTG | Used to amplify *S. enterica smvA* or *smvAR* |
| SalTy SmvA Prom F XbaI | TATATCTAGACATAGCGTGAAGCCGTGCAC | Used to amplify *S. enterica smvA* |
| SalTy SmvR R XbaI | TAGGCTAGATTACGCAAAGAGTTCAAGCG | Used to amplify *S. enterica smvAR* |

Supplementary Table 4 - List of primers used in the study

## Supplementary Methods

### Molecular Docking & Generating the SmvA Complexes

AutoDock SMINA (1), which uses the AutoDock Vina scoring function by default, was used for the blind molecular docking of the compounds to SmvA structure for finding the best binding site in the target by exploring all probable binding cavities of the transporter. SMINA was performed with default settings, which samples nine ligand conformations using the Vina docking routine of stochastic sampling. Then GOLD molecular docking (2,3) was applied for the docking of the compounds to the SMINA-located best binding site of the efflux pump for performing flexible molecular docking. Based on the fitness function scores and ligand binding positions, the best-docked poses for the compounds were selected. The higher fitness function score of poses, generated using the GOLD program that has the more negative GOLD fitness energy value, reveals the best-docked pose for each compound.

The GOLD molecular docking procedure was performed by applying the GOLD protocol (3). The Genetic algorithm (GA) was used in GOLD ligand docking software to examine the ligand conformational flexibility along with the partial flexibility of the protein (4) thoroughly. The maximum number of runs for the ligand was set to 20, and in each run, a population size of 100 with 100,000 operations was employed. The number of islands was 5, and the niche size of 2 was considered. The default cut-off value for hydrogen bonds was set to 2.5Å (dH-X), and for the van-der-Waals distance, it was 4.0Å. The GA docking was terminated when the top solutions attained the root-mean-square deviations (RMSD) values within 1.5 A˚.

### Molecular Dynamics (MD) Simulations

After performing the molecular docking, 100 ns independent MD simulations were performed for ligand-bound complexes and the ligand-free proteins. All the MD simulations were carried out using the AMBER 16.0 package, and MMPBSA/MM-GBSA energy calculations were used as post-processing analysis. Each system was solvated by using an octahedral box of TIP3P water molecules. Periodic boundary conditions and the particle-mesh Ewald method were employed in the simulations (5). Particle-mesh Ewald method enabled us to calculate the 'infinite' electrostatics without truncating the parameters. During each of the simulations run, all bonds in which the hydrogen atom was present were considered fixed, and all other bonds were constrained to their equilibrium values by

applying the SHAKE algorithm (6). The force fields parameters for the compounds were generated by using the ANTECHAMBER module of the AMBER program.

A cut-off radius of non-covalent interactions was set to 12 Å for the proteins and complexes. Each minimisation and equilibration phases was performed in two stages. In the first stage, ions and all water molecules were minimised for 2500 cycles of steepest descent followed by 2500 cycles of conjugate gradient minimisation. Afterwards, the whole system was minimised for a total of 5000 cycles without restraint wherein 2500 cycles of steepest descent were followed by 2500 cycles of conjugate gradient minimization. In the second stage, the systems were equilibrated for 500 ps while the temperature was raised from 0 to 300 K, and then equilibration was performed without a restraint for 1 ns while the temperature was kept at 300 K. Sampling of reasonable configurations was conducted by running 100 ns simulations with a 2 fs time step at 300 K and 1 atm pressure. A constant temperature was maintained by applying the Langevin algorithm while the pressure was controlled by the isotropic position scaling protocol used in AMBER (7).

**MM-PBSA/MM-GBSA Energy Calculations**

Twenty snapshots were collected from the last 200 ps of simulations of protein-ligand complexes for post-processing analysis. The gas-phase interaction energy between the protein and the ligand, ΔEMM, is the sum of electrostatic (ΔEELE), internal (ΔEINT) and van der Waals (ΔEVDW) interaction energies. The solvation free energy ΔGsol is the sum of polar (ΔGPB) and non-polar (ΔGSA) parts. The ΔGPB term was calculated by solving the finite-difference Poisson-Boltzmann equation using the internal PBSA program. The SCALE value was set to 5. The Parse radii were employed for all atoms (8). The solvent probe radius was set at 1.4 Å (with the radii in the prmtop files). MM-PBSA running was performed with the PBSA module (PROC=2). The value of the exterior dielectric constant was set at 80, and the solute dielectric constant was set at 1 (9). The nonpolar contribution was determined on the basis of the solvent accessible surface area (SASA) using the LCPO method (10), $\Delta GSA = 0.04356 \times \Delta SASA$ and CAVITY-OFFSET set at -1.008.

$\Delta G_{bind} = \Delta G_{PB/GB} - T\Delta S$

$\Delta G_{PB/GB} = \Delta E_{GAS(MM)} + \Delta E_{PB/GBsol}$

$\Delta E_{GAS(MM)} = \Delta E_{internal} + \Delta E_{electrostatic} + \Delta E_{vdw}$

$\Delta E_{PB/GBsol} = \Delta E_{PB/GBsur} + \Delta E_{PB/GBcal}$

In the MM-GBSA calculations, like the MM-PBSA calculations, the gas-phase interaction energy (ΔEGAS(MM)) and the non-polar and polar (ΔEPB/GBsol) parts of the solvation energy was calculated. The electrostatic solvation energy (ΔGGB) was calculated using GB models (11). A value of 80 was used for the exterior dielectric constant, and a value of 1 was used for the solute dielectric constant. The relative binding free energies were calculated using both the MM-PBSA and the MM-GBSA methods.

**References**

(1) Koes, D.R.; Baumgartner, M.P.; Camacho, C.J. Lessons learned in empirical scoring with smina from the CSAR 2011 benchmarking exercise. J Chem Inf Model 2013, 53, 1893-904.

(2) Jones, G.; Willett, P.; Glen, R.C. Molecular recognition of receptor sites using a genetic algorithm with a description of desolvation. J Mol Biol 1995, 245, 43-53.

(3) Jones, G.; Willett, P.; Glen, R.C.; Leach, A.R.; Taylor, R. Development and validation of a genetic algorithm for flexible docking. J Mol Biol 1997, 267, 727-48.

(4) Nissink, J.W.; Murray, C.; Hartshorn, M.; Verdonk, M.L.; Cole, J.C.; Taylor, R. A new test set for validating predictions of protein-ligand interaction. Proteins 2002, 49, 457-71.

(5) Darden, T.Y., D.; Pedersen, L. Particle Mesh Ewald - an N.Log(N) Method for Ewald Sums in Large Systems. J Chem Phys 1993, 98, 4.

(6) Ryckaert, J.P.C., G.; Berendsen, H. J. C. Numerical-Integration of Cartesian Equations of Motion of a System with Constraints - Molecular-Dynamics of N-Alkanes. J Comput Phys 1977, 23, 15.

(7) Case, D.A.; Cheatham, T.E.; Darden, T.; Gohlke, H.; Luo, R.; Merz, K.M.; Onufriev, A.; Simmerling, C.; Wang, B.; Woods, R.J. The Amber biomolecular simulation programs. J Comput Chem 2005, 26 1668-88.

(8) Sitkoff, D.; Sharp, K.A.; Honig, B. Accurate Calculation of Hydration Free-Energies Using
molecular dynamics and a continuum solvent model. J Mol Biol 2000, 303, 16.

(9) Wang, W.K., P. A. Free energy calculations on dimer stability of the HIV protease using molecular dynamics and a continuum solvent model. J Mol Biol 2000, 303, 16.

(10) Weiser, J.; Shenkin, P.S.; Still, W.C. Approximate atomic surfaces from linear combinations of pairwise overlaps (LCPO). J Comput Chem 1999, 20, 217-30.

(11) Tsui, V.; Case, D.A. Theory and applications of the generalized Born solvation model in macromolecular simulations. *Biopolymers* 2000, *56*, 275-91.
